# Deep Learning Techniques for Speech Emotion Recognition, from Databases to Models

**DOI:** 10.3390/s21041249

**Published:** 2021-02-10

**Authors:** Babak Joze Abbaschian, Daniel Sierra-Sosa, Adel Elmaghraby

**Affiliations:** Computer Science and Engineering Department, University of Louisville, Louisville, KY 40292, USA; d.sierrasosa@louisville.edu (D.S.-S.); adel@louisville.edu (A.E.)

**Keywords:** attention mechanism, autoencoders, CNN, deep learning, emotional speech database, GAN, LSTM, machine learning, speech emotion recognition

## Abstract

The advancements in neural networks and the on-demand need for accurate and near real-time Speech Emotion Recognition (SER) in human–computer interactions make it mandatory to compare available methods and databases in SER to achieve feasible solutions and a firmer understanding of this open-ended problem. The current study reviews deep learning approaches for SER with available datasets, followed by conventional machine learning techniques for speech emotion recognition. Ultimately, we present a multi-aspect comparison between practical neural network approaches in speech emotion recognition. The goal of this study is to provide a survey of the field of discrete speech emotion recognition.

## 1. Introduction

Speech emotion recognition is the task of recognizing emotions from speech signals; this is very important in advancing human–computer interaction:

Human computer interaction is characterized as consisting of five major areas of study: research into interactional hardware and software, research into matching models, research at the task level, research into design, and research into organizational impact [1].

Understanding one’s feelings at the time of communication is constructive in comprehending the conversation and responding appropriately. Currently, this part of human–computer interaction has not yet entirely been solved, and except for a limited number of applications, there is no general solution to this problem [2,3].

Along with all major problems in machine learning, SER has started to gain an advantage from the tools made available by deep learning. Before the extensive employment of deep learning, SER was relying on methods like hidden Markov models (HMM), Gaussian mixture models (GMM), and support vector machines (SVM) along with lots of preprocessing and precise feature engineering [4,5,6]. However, with deep learning making up most of the new literature, the results are going up from around 70% accuracy to the upper 90s in controlled environments.

Automatic SER helps smart speakers and virtual assistants to understand their users better, especially when they recognize dubious meaning words. For example, the term “really” can be used to question a fact or emphasize and stress out a statement in both positive and negative ways. Read the following sentences in different ways: “I really liked having that tool.” The same application can help translate from one language to another, especially as other languages have different ways of projecting emotions through speech [7]. SER is also beneficial in online interactive tutorials and courses. Understanding the student’s emotional state will help the machine decide how to present the rest of the course contents [8]. Speech emotion recognition can also be very instrumental in vehicles’ safety features. It can recognize the driver’s state of mind and help prevent accidents and disasters [8]. Another related application is in therapy sessions; by employing SER, therapists will understand their patients’ state and possibly underlying hidden emotions as well [9]. It has been proven that in stressful and noisy environments like aircraft cockpits, the application of SER can significantly help to increase the performance of automatic speech recognition systems [10]. The service industry and e-commerce can utilize speech emotion recognition in call centers to give early alerts to customer service and supervisors of the caller’s state of mind [11]. In addition, speech emotion recognition has been suggested to be implemented in interactive movies to understand viewers’ emotions. The interactive film could then go along different routes and have different endings [12].

To train machine learning algorithms to classify emotions, we need to have training datasets. For SER tasks, there are generally three types of training datasets, natural, semi-natural, and simulated. The natural datasets are extracted from available videos and audios, either broadcasted on TV or online. There are also databases from call centers and similar environments. Semi-natural datasets are made by defining a scenario for professional voice actors and asking them to play them. The third and most widely used type, the simulated datasets, are similar to semi-naturals. The difference is that the voice actors are acting the same sentences with different emotions.

Traditionally SER used to follow the steps of automatic speech recognition (ASR), and methods based on HMMS, GMMs, and SVMs were widespread [5,6,13,14,15,16,17]. Those approaches needed lots of feature engineering and any changes in the features usually required restructuring the entire architecture of the method. However, lately, by the development of deep learning tools and processes, solutions for SER can be changed as well. There is a lot of effort and research on employing these algorithms to recognize emotions from the speech [18,19,20,21,22,23,24,25,26,27]. In addition to deep learning, more recently, along with improvements in recurrent neural networks and the use of long short-term memory (LSTM) networks, autoencoders, and generative adversarial models, there has been a wave of studies on SER using these techniques [28,29,30,31,32,33] to solve the problem.

The rest of the paper is organized as follows: In Section 2, we define SER, and in Section 3, we present some related studies. In Section 4, we provide a review of existing emotional speech datasets, and Section 5 is where we review several traditional and deep learning methods used in SER. Finally, in the last chapter, we discuss and conclude our work while proposing direction for future actions in SER.

## 2. Speech Emotion Recognition Definition

To be able to comprehend the messages we receive, we need to complement what we hear with other signals from the interlocutor. One of the signals is understanding the emotions of our collocutor while communicating. Understanding the feelings in correlation with the message comprehended will be an essential key to a fruitful conversation [34]. Along with all the benefits humans would gain of understanding emotions, it is evident that in human–computer interaction, we would be able to gain a lot as well. In recent years, there are many studies, efforts, and even competitions focused on building means and methods to create such an understanding for computers [35,36,37,38].

To be able to classify emotions using computer algorithms, we need to have a mathematical model describing them. The classical approach defined by psychologists is based on three measures that create a three-dimensional space that describes all the emotions. These measures or dimensions are pleasure, arousal, and dominance [39,40]. A combination of these qualities will create a vector that will be in one of the defined emotion territories, and based on that, we can report the most relevant emotion [41].

Using pleasure, arousal, and dominance, we can describe almost any emotion, but such a deterministic system will be very complex to implement for machine learning. Therefore, in machine learning studies, typically, we use statistical models and cluster samples into one of the named qualitative emotions such as anger, happiness, sadness, and so forth. To be able to classify and cluster any of the mentioned emotions, we need to model them using features extracted from the speech; this is usually done by extracting different categories of prosody, voice quality, and spectral features [42].

Any of these categories have benefits in classifying some emotions and weaknesses in detecting others. The prosody features usually focused on fundamental frequency (F_0_), speaking rate, duration, and intensity, are not able to confidently differentiate angry and happy emotions from each other [42]. Voice quality features are usually dominant in the detection of emotions of the same speaker. Still, they differ from speaker to speaker, and it is making them hard to be used in a speaker-independent setting [43]. Spectral features have been extensively analyzed to derive emotions from speech. The immediate advantage that they have compared to prosody features is that they can confidently distinguish angry from happy. However, an area of concern is that the magnitude and shift of the formants for the same emotions vary across different vowels, and this would add more complexity to an emotion recognition system, and it needs to be speech content-aware [44].

For every one of those feature categories, as mentioned earlier, there are various standard feature representations. Prosody features are usually being shown by F0 and measures related to speaking rate [45], and spectral features are generally being described using one of the cepstrum-based representations available. Commonly, Mel-frequency cepstral coefficients (MFCC) or linear prediction cepstral coefficients (LPCC) are used, and in some studies, spectral features, formants, and other information are used as well [46]. Voice quality features are usually described by normalized amplitude quotient (NAQ), shimmer, and jitter [47].

There are two major approaches in SER, either recognizing based on the three dimensions of emotions or recognition based on statistical pattern recognition techniques for the qualitative named emotions. For the first approach, we calculate degrees of correlation between the given signal and passion, arousal, and dominance, and then using a hierarchical classifier, the complex emotion is determined. The second group is done using statistical pattern recognition methods, such as the Gaussian mixture model (GMM) [14], support vector machine (SVM) [5], hidden Markov model (HMM) [15], artificial neural network (ANN) [13], deep neural network (DNN) [24], and genetic algorithm (GA) [48].

## 3. Related Work

Due to the importance of SER in human–computer interaction and the development of artificial intelligence systems, there are multiple other recent publications and surveys on SER. In this section, we review the most recent studies related to the current work.

In 2018, Swain et al. [49] reviewed studies between 2000 and 2017 on SER systems based on three perspectives: database, feature extraction, and classifiers. The research has an extensive section on databases and feature extraction; however, only traditional machine learning methods have been considered as classifying tool, and the authors are regretting neural networks and deep learning approaches.

A year after, Khalil et al. [50] reviewed discrete approaches in SER using deep learning. Several deep learning approaches, including deep neural networks (DNNs), convolutional neural networks (CNNs), recurrent neural networks (RNNs), and autoencoder, have been mentioned along with some of their limitations and strengths in the study. However, the research is not addressing the accessible approaches to overcome weaknesses.

Very recently, Anjali et al. [51] published a review as a summary of speech emotion detection methods. A brief discussion of various features used in speech emotion recognition and review of methods used for this purpose from 2009 to 2018 has been provided in the re-search. The drawback of the paper is the depth of analysis. Yet, it can be considered a start point.

In 2020, Basu et al. [52] published a brief review on the importance of speech emotion datasets and features, noise reduction; ultimately, they analyze the significance of different classification approaches, including SVM and HMM. The strength of the research is the identification of several features related to speech emotion recognition; however, its weakness is the leak of more modern methods’ investigation and briefly mentions convolutional and recurrent neural networks as a deep learning method.

Ultimately, a survey by Akcay et al. [53] provides a relatively comprehensive study on databases, features, classifiers, and emotion models. The research is also reviewing machine learning techniques for classification enhancement. However, no comparable results from different methods have been reported except for initial results from their original papers.

In contributions to the other published surveys, this research provides a thorough study of significant databases and deep learning discrete approaches in SER. The reason for not focusing on the other older techniques is recent progress in neural networks and, more specifically, deep learning. Based on the best of our knowledge, this study is the first survey in SER focusing on deep learning along with unified experimental results that proposes approaches to enhance the available methods’ results.

## 4. Emotional Speech Databases

For every machine learning task, we need to have a training set of samples; SER is not different from the rest. The process of creating a training dataset for SER needs human agents to label the samples by hand, and different people perceive emotions differently. For example, one might tag an emotional voice as angry whilst the other perceives it as excited. This ambiguity means to label the samples we must have more than one agent reviewing each and then having a system to choose the labels for available samples confidently. There are three types of databases specifically designed for speech emotion recognition, simulated, semi-natural, and natural speech collections. The simulated datasets are created by trained speakers reading the same text with different emotions [54]. Semi-natural collections are made by asking people or actors to read a scenario containing different emotions. Moreover, natural datasets are extracted from TV shows, YouTube videos, call centers, and such, and then labeled the emotions by human listeners [54].

Simulated data sets such as EMO-DB (German) [55], DES (Danish) [56], RAVDESS [57], TESS [58], and CREMA-D [59] are standardized collections of emotions, which makes comparing results very easy. Although their numbers of distinct emotions are significant, as they have synthesized emotions, they tend to have overfitted models around emotions slightly different than what is happening in day-to-day conversations.

Semi-natural collections of emotions include IEMOCAP [60], Belfast [61], and NIMITEK [62]. This group has the advantage of being very similar to the natural utterances of speech. However, even though they are based on scenarios and the speech is happening in a contextual setting, they are artificially created emotions, especially when speakers know that they are being recorded for analysis reasons. Additionally, due to the limitations of the situations in scenarios, they have a limited number of emotions in comparison to the previous group.

The last group is the natural corpora of emotional speech databases such as VAM [39], AIBO [63], and call center data [45,64]. These are entirely natural, and they can be safely used to model emotion recognition systems without hesitation about being artificially made. However, modeling and detection of the emotions with this type of datasets can be complicated due to the continuousness of emotions and their dynamic variation during the course of the speech, and the existence of concurrent emotions together, and the presence of background noise. Additionally, because the sources of the data were limited, the number of different emotions found in these corpora is limited. Moreover, there can be potential copyright and privacy issues arise using this type of corpora. The major challenge in using this type of dataset is the need for noise reduction.

Earlier examples of databases for emotional speech used to contain a limited number of samples with a limited number of actors, but newer databases tend to create a larger number of samples and a wider range of speakers. Table 1 shows a brief comparison of different types of databases, as discussed above, pointing out the differences in the features of every database and some examples of each type. In the following, we will review EMO-DB and DES, commonly used in SER research; three recent and openly available English simulated databases; IEMOCAP, a semi-natural database; and VAM, a natural dataset used for speech emotion recognition.

### 4.1. Berlin Database of Emotional Speech (EMO-DB)

The Berlin Database of Emotional Speech (EMO-DB) [55] is one of the most widely used datasets for speech emotion recognition. It is a simulated dataset composed of 10 German sentences, five short sentences, and five long sentences. Ten speakers, five females, and five males were employed to create the dataset. Each one of the speakers had expressed ten sentences, five long and five short, with different emotions.

Along with the recording of the audio signal, to be able to extract prosodic and voice quality more precisely, the electroglottograms were also recorded along with the voices.

The whole dataset is containing 700 samples, of 10 sentences acted with seven emotions. The emotions chosen for this dataset were neutral, anger, fear, joy, sadness, disgust, and boredom.

### 4.2. Danish Emotional Speech Database (DES)

For the Danish emotional speech database [56], four actors, two males and two females, were employed. Each one of the actors had read two single words, nine sentences, and two long passages of fluent speech with five emotions of neutral, surprise, happiness, sadness, and anger each. Additionally, some of the actors had read extra sentences and passages. All the recordings then were played for 20 listeners and were ranked and labeled.

### 4.3. The Ryerson Audio-Visual Database of Emotional Speech and Song (RAVDESS)

The RAVDESS [57] is a dataset consisting of happy, sad, angry, fearful, surprised, disgusted, calm, and neutral emotions performed by 24 actors, 104 sentences each actor [57]. Totaling 2496 clips, RAVDESS is very rich in variations of the samples; also, every emotion is performed in two different intensity and both with a normal voice and singing voice. This is one of the crucial features of RAVDESS, and only a few numbers of data sets can claim to have such a feature. Moreover, RAVDESS is among the few datasets that contain, North American English accent, and this could be important in cases that the American English accent makes the evaluation test set.

### 4.4. Toronto Emotional Speech Set (TESS)

Toronto Emotional Speech Set (TESS) [58] is an acted dataset primarily developed for analyzing the effect of age on the ability to recognize emotions. This dataset is all comprised of two female actors, about 60 and 20 years old. Each actor has simulated seven emotions for 200 neutral sentences. Emotions in this dataset are: angry, pleasantly surprised, disgusted, happy, sad, fearful, and neutral. To label the dataset, 56 undergraduate students were asked to identify emotions from the sentences. After the identification task, the sentences with over 66% confidence have been selected to be in the dataset.

### 4.5. Crowd-Sourced Emotional Multimodal Actors Dataset (CREMA-D)

CREMA-D [59] is a dataset made for multimodal emotion recognition tasks. The data set covers six emotions of anger, happiness, sadness, fear, disgust, and neutral and consists of 7442 samples acted by 91 actors from a wide range of ethnic backgrounds. The labels were generated by crowd-sourcing of 2443 raters. Each sample in this dataset has two ratings, one for the category of emotions and the second for its intensity. Labeling in this dataset was done by aggregation of 223,260 individual ratings through 2443 individual raters. The labels were cross verified with the initially intended labels, and using Krippendorff’s alpha measure, the good samples were chosen. In this dataset, they tried to have a similar number of samples in each class.

### 4.6. Interactive Emotional Dyadic Motion Capture Database (IEMOCAP)

IEMOCAP [60] is a semi-natural English audiovisual dataset acted by ten speakers, five males and five females with 1150 Utterances. The original emotion set in database scenarios was consisting of anger, happiness, sadness, and frustration. However, later they labeled data with four additional categories of disgust, fear, excitement, and surprise. They also included the labeled data with continuous qualities of valence, activation, and dominance. The dataset is not free to use and is available with a license.

This semi-natural dyadic database has more naturally emitted emotions than the simulated databases. With 5 min average duration of each dialog, it will be one of the very well-suited datasets for deep learning applications.

### 4.7. Vera am Mittag Database (VAM)

VAM [39] dataset is a natural audiovisual dataset based on the dialogues in the German TV talk show Vera am Mittag. It consists of valence, activation, and dominance, useful for dimensional speech emotion recognition. The audio part of the dataset was performed by 47 speakers from the show with 1018 audio utterances.

Being a natural dataset makes it unique in containing the utmost real emotions. Still, at the same time, the emotions are not focused and contained, and in a dialog, you would see a fluid transition between many emotions. However, they separated the sentences as their utterances and labeled them with an emotion.

**Figure 1:**
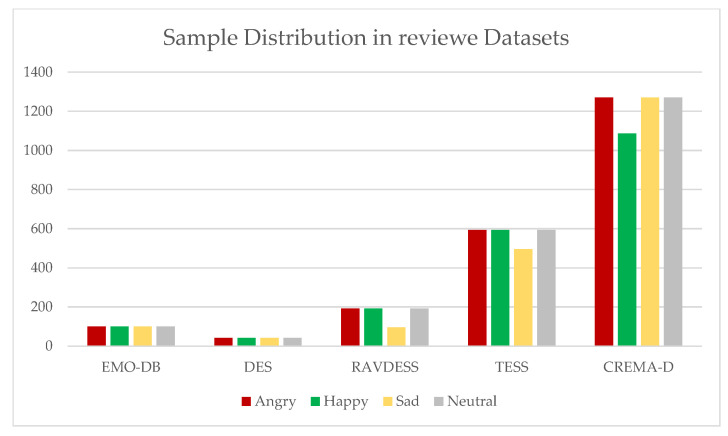
Comparison of the distribution of similar emotions from the reviewed simulated datasets.

Table 2 shows an aggregated view of the reviewed datasets and their features. In this table, the numbers of emotions, samples, and speakers, and the average length of samples in each database have been compared. As can be seen, only four emotions of anger, happiness, sadness, and neutral are shared in between of all the datasets. In addition, Figure 1, portrays the distribution of common emotions in the simulated reviewed datasets, as you can see, the newer datasets have more samples and more actors. We have not included IEMOCAP in this figure as semi-natural, and natural datasets do not have a balanced number of emotions. IEMOCAP with 3220 neutral samples compared to 805 angry samples was going to make the rest of the figure unreadable.

## 5. Emotion Recognition Methods

There are many methods and algorithms used for the task of emotion recognition from speech. Each one of these methods is trying to solve the problem from a specific angle and have advantages and shortcomings. Historically most of the methods for speech emotion recognition were based on classic machine learning algorithms, specifically HMM and SVM. However, in recent years, the trend is shifted towards deep learning-based methods.

In this chapter, we will first briefly review the traditional methods. Then we will study some of the newer research and solutions proposed for the problem of Speech Emotion Recognition.

### 5.1. Traditional Methods

In earlier efforts to recognize emotions from the speech signal, almost all the implementations were based on machine learning and signal processing methods; following the same path of automatic speech recognition, there were many implementations based on SVM, GMM, and HMMs. They were implementing frameworks based on machine learning algorithms that needed extensive feature engineering and a deep understanding of the subject matter to be able to infer the features helping the most to bring them into the calculations. In this part, we will review two of the methods based on HMM and SVM to build a basis for the next generation of algorithms.

#### 5.1.1. Hidden Markov Models (HMM)

HMMs have been playing a prominent role in speech recognition since the 1960s [65]. They are built to create a flexible model conforming to the speech’s temporal features, thus classifying minute variations in an audio signal. Naturally, HMMs were one of the first choices to try in SER. Some researchers have tried to solve the problem using frequency representations such as MFCCs and LPCCs [15], and studies focused on low-level descriptors such as energy content and pitch [66].

There also are studies that use more emotion recognition tailored features, such as prosodic features in Lin et al.’s work [6]. They considered temporal dynamics and spectral characteristics using HMM and specified features in speech emotion and rate analysis. Their work has produced comparatively good classification results in addition to reinforcing the hypothesis that Mel sub-band energies and temporal dynamics of the fundamental frequency are vital indicators in speech’s emotional content.

#### 5.1.2. Support Vector Machine (SVM)

Support Vector Machines, as one of the well-known methods, is used as a classifier for emotion recognition. Generally, SVM is utilized for classification and regression purposes. They are performing classification by constructing an N-dimensional hyperplane, which separates the data into categories optimally. A linear or nonlinear separating surface obtains the classification in the input feature space of the dataset. The main idea in SVM is using a kernel function to transform the original input set to high-dimensional feature space and then achieve optimum classification in this new feature space. LIBSVM [16] is a widely used tool for SVM classification and regression developed by C. J. Lin. Radial Basis Function (RBF) kernel is used in the training phase. The advantage of using the RBF kernel is that it restricts training data to lie in specified boundaries. The RBF is a nonlinear kernel that maps samples into a higher dimensional space with less numerical difficulties than the polynomial kernel.

In the literature, SVM has been applied to the Berlin emotion database of German language [17], and from the speech files in .wav format, MFCC and MEDC features were extracted. The result of this research proves that such a system is speaker-independent and text-independent, and using RBF and polynomial kernel function, LIBSVM’s results were 93.75% and 96.25%, respectively.

### 5.2. Neural Networks and Deep Learning

Artificial neural networks were up-and-coming in the early 80s, and there were lots of research trying to use these newly emerging techniques. Still, due to lack of process speed and memory requirements, they were not able to have deeper architectures than one or two layers, thus not very impressive results. By improvements in memory, CPU, and GPU power of the computers, now more sophisticated and deeper architectures are possible to design and test. Moreover, this has resulted in lots of new methods and approaches based on a variety of deep neural networks in various research fields from medicine, to finance, cybersecurity, and root cause analysis [67,68,69,70,71,72]. Among these variants, artificial neural networks, convolutional networks, and LSTM networks are more pandemic, and especially in speech emotion recognition and sentiment analysis [37,70,73], are the new replacements for HMM and SVM architectures. In the following part, we are going to review some of the recent approaches.

#### 5.2.1. Artificial Neural Networks

In machine learning and computer science, many problems cannot be converted to a straight forward algorithm to be solved. The solution to those problems needs to be dynamically adapted for every situation the algorithm is applied. The adaptation is an essential feature of human brains, but in the case of computers, there is no easy way of creating an adaptive code. Our brain has generalization capability, which helps it to reason inductively, which is the very first step of learning. ANN is a compelling solution for adaptive learning. ANNs are capable of learning complex nonlinear relations between inputs and desirable outputs, these systems are being used widely in almost every area of machine learning nowadays, and SER is not an exception [74].

In their research, Shaw et al. [18] have created a recognition system using artificial neural networks to recognize four classes of happy, angry, sad, and neutral. To implement their system, they incorporate both prosodic and spectral features for the classification task. Their network has an input layer, one hidden layer, and the output of the four classes. The result of their work is 81% of overall accuracy for all their classes.

Later in 2018, Darekar and Dhande [19] have introduced a system based on artificial neural networks, the first extract NMF analysis, Pitch Analysis, and Cepstrum features; then, they reduce their dimensionality applying PCA to their feature vectors. They then feed their features to an artificial neural network introduced by Bhatnagar and Gupta 2017 [20], called NARX Double Layer, which is an ANN with two hidden layers. To train their network, they have adopted a PSO Feedforward algorithm, which helps to reach optimal weights faster than gradient descent. They have applied their model on the RAVDESS dataset and have shown that they gain 10.85% accuracy over the baseline method applied on RAVDESS. They suggest that keeping the PSO Feedforward and replacing ANN with a better classifier, the results would be further improved.

Implementation and training of ANNs are faster than the rest of the neural network-based methods, but a single layer ANN cannot solve very complex and nonlinear problems, that is the boundary that ANN stops. Thus, essentially, they may be fast, but they are limited in their capabilities.

#### 5.2.2. Deep Learning

Deep learning is an unofficial name for a group of machine learning algorithms, usually consisted of multi-layer neural networks. Yan Lecun in 1988 introduced backpropagation in multi-layer convolutional neural networks and made it possible to create deep structures. However, mostly because of the process limitations, they did not become a mainstream tool in machine learning until recently. One of the most famous examples that sparked widespread use of deep learning was the introduction of AlexNet in 2012 by Krizhevsky et al. [75], which was a multi-layer convolutional neural network trained on ImageNet in 2010 to recognize 1000 different classes and achieved excellent results. After that, there was a surge of different deep architectures. Following, we will review some of the most recent applications of deep learning on Speech Emotion Recognition.

In 2011, Stuhlsatz et al. [22] introduced a system based on deep neural networks for recognizing acoustic emotions, GerDA (generalized discriminant analysis). Their generalized discriminant analysis was a generalization of linear discriminant analysis, which maximizes a Fisher discriminant criterion over a nonlinear function space defined by a deep neural network. They have applied their method to 9 different most frequently used databases, leaving one speaker (or group) out. Comparing their results with standard SVM methods, they were able to improve the accuracy and the results.

Later in 2014, Han et al. [23] have used a deep neural network to create a probability distribution for different emotions given every segment. They also have used a Shallow single neural network to identify emotions from utterance features, their single hidden layer network, ELM (extreme learning machine), which can achieve outstanding classification results when the training set is small. To be able to recognize emotions, they have combined their segment level distributions from DNN to create utterance level features, and they have given those features to ELM to classify the emotions. To compare their proposed method with others, they have used a standard HMM-based recognizer, a standard SVM based system, a DNN-HMM recognizer, and a DNN-SVM based system. They have shown that the accuracy of their approach is considerably (5–20%) higher than all the methods compared.

In the same year, Amer et al. [24] used nonlinear restricted Boltzmann machines (RBM). This approach was used to create a conditional RBM to be able to build a distribution model for the features to form a generative classifier classifying short-term temporal representations of affections in speech. Then they staged their deep network with a conditional random field (CRF) classifier implemented with a shallow neural network to be able to create long-term utterance level decisions on the emotions the recognize in speech.

They then tested their system with three different databases (AVEC, VAM, and SPD) and seen a moderate improvement compared to a range of other networks. However, in many instances, especially in tests on the AVEC dataset, HMM-CRF models outperformed their system by tenths of a percent, which they justified with their system speed and lack of long-term labeled samples [24].

Lately, Tiwari et al. [76] address the noise robustness of SER in the presence of additive noise by employing an utterance level parametric generative noise model. Their deep neural network framework is useful for defeating unseen noise since the generated noise can cover the entire noise space in the Mel filter bank energy domain. The proposed method aimed to be successful in data augmentation scenarios such as a limited number of samples in EMO-DB; however, it needed to be improved to achieve such a promising goal.

Deep neural networks can be defined as any network with more than one hidden layer. With this definition, they cover all the deep structures such as convolutional, long short-term memory, and generative adversarial networks. However, in this text, we have separated deep artificial neural networks in the sense of only using linear, fully connected layers from other deep networks incorporating any additional nonlinear layer.

Thus, in the sense of our definition, deep artificial neural networks have better capabilities in modeling problems than their single-layer siblings. However, their modeling is still limited to nonlinear polynomial functions, and the cost of such ability is the exponential increase in the number of the variables to train; hence more process and more memory.

#### 5.2.3. Convolutional Neural Networks

Convolutional neural networks (CNNs) or shift invariant artificial neural networks (SIANNs) are particular types of neural networks that, in their hidden layer they have different filters or regions that respond to a specific feature of the input signal. Their design is based on the research by Hubel and Wiesel in 1968 [77], which introduces the visual neural cortex as a spatially specialized structure, in which every region responds to a specific characteristic of the input signal. One positive perspective of CNNs is the ability to learn features from high-dimensional input data; however, it also learns features from small variations and distortion appearance that leads to the large storage requirement at the time of development. Hence, in CNNs, there usually exists a layer of convolution followed by a downsampling mechanism. The convolution layer has various filter banks, in which their weights will be tuned through often backpropagation, Weng et al. 1993 [78].

In their research, D. Bertero and P. Fung [21] have introduced a convolutional neural network that is capable of detecting angry, happy, and sad emotions with a 66.1% accuracy rate. They have compared their blind trained network outcome with a baseline Feature-based SVM. To be able to train and test their method, they have used a corpus of TED talks, labeled by students and crowd sourced. They have implemented their CNN using the Theano toolkit. For comparison, they have trained a linear SVM with a feature set from the INTERSPEECH 2009 emotion challenge.

They reported that their CNN network, with less than a couple hundred milliseconds response time, can detect three classes of angry, happy, and sad with a 66.1% accuracy rate. They showed that their neural activity is concentrated around the fundamental frequencies most correlated to the emotions.

In 2020, Mekruksavanich et al. [79] experiment with a one-dimensional convolutional neural network and report the accuracy of 96.60% in classifying negative emotions from Tai language datasets. In their study, the developed method has been applied to SAVEE, RAVDESS, TESS, and CREMA-D and Tai datasets.

#### 5.2.4. Deep Convolutional Neural Networks

Deep convolutional networks usually consist of multiple layers of convolution nodes, followed by one or more fully connected layers to finish the classification task. In SER, there are many efforts on deep convolutional neural networks, which we will review some of the most recent ones in the following part.

Harar et al. [26] have proposed a method based on a deep neural network containing convolutional pulling and fully connected layers. They have implemented their system on the Berlin Database of Emotional Speech. To compare to previous research, they have limited their classes to angry, neutral, and sad. In their system, they have removed silence from their signals and then divided the files into 20 ms chunks with no overlap. In their network, before any feature selection, they have six layers of convolution with succeeding dropout layers with *p* values of 0.1 and after that follows a lattice of two parallel feature selectors and then series of fully connected layers.

The segment accuracy of their system was 77.51%, but the file level accuracy was 96.97%, with a 69.55% confidence rate. Although the system’s accuracy on file level was high, in real scenarios, there is no sign to point a chunk of speech, and the system needs to improve on independent detection.

Zhang et al. [25] have developed an Emotion recognition system based on a deep convolutional neural network designed for the ImageNet LSVRC-2010 contest. This network, AlexNet, is also pretrained with a dataset of 1.2 million images, then fine-tuned using samples that they had from EMO-DB. Using this system, they can recognize three classes of emotions (angry, sad, and happy) plus a neutral category. Moreover, they have demonstrated that their system can have accuracies over 80% with EMO-DB, about 20% more than the baseline SVM standard. They have also applied their method to 3 other databases (RML, eNterface05, and Baum-1s) and were able to get results higher than the baseline method.

In their system, the focus was on how automatic feature selection in deep convolutional neural networks can outperform feature selection in shallow convolutional networks and Statistical model-based methods like GMM and HMM. One of the essential features used in this system was the use of the discriminant temporal pyramid matching (DTPM) strategy, which helps in concatenating the learned segment level feature to form an utterance level feature representation. Deep convolutional neural networks are potent in modeling the smallest transient attributes of the signal. However, this capability comes with the cost of exponentially more variables to tune, and this means more samples are needed to train the system. In the case of image applications, these networks get trained by millions of samples. However, in SER, usually, the numbers of the samples are limited to thousands. Additionally, this makes solutions based on deep convolutional networks more prone to overfitting.

#### 5.2.5. LSTM Networks

Recurrent neural networks can learn and react to the temporal event without changing the slowly shaped weights thanks to their feedback connection, forming short-term activations for recent events. This feature can be beneficial in case of applications that time is an essential feature, like Speech Processing, music composition, and video description. However, as they are trained using Back Propagation through Time, error signals flowing backward in time can either become bigger and bigger or vanish depending on the size of the weights. This will create either oscillating weights or makes the network to be slow to train and converge [80].

To be able to incorporate the short-term adaptation of RNNs and avoid the problems above, Hochreiter and Schmidhuber [80] introduced a new architecture called Long Short-Term Memory in 1997. LSTM networks are capable of bridging time intervals bigger than 1000 steps, even if input sequences are incompressible and noisy. They are incorporating a gradient-based algorithm enforcing constant error flow through individual units, specially designed to handle the short-term; thus, they can truncate the gradient computations at a definable point without affecting the long-term activations.

In recent years, LSTM networks were becoming in the center of attention for many applications involving time series events. Speech Processing and especially speech emotion recognition are two of these applications.

An early proposal for using LSTM networks in 2013 was in the work of Martin Wöllmer et al. [28]. They have proposed a multimodal LSTM based classification network, exploiting acoustic, linguistic, and visual information. In their study, they have compared both unidirectional and bidirectional LSTM networks. They also have compared their proposed results with the AVEC 2011 Audio/Visual Emotion Challenge [62]. In this research, they extract 1941 audio features composed of Prosodic, Spectral, and Voice quality features, linguistic word-level content, and all the video features extracted by applying the Viola-Jones method, segmented optical flow, and head tilt. Then all the features are being fed to a unidirectional and a bidirectional LSTM network.

Later in 2016, Trigeorgis et al. [29] have proposed a context-aware system for end-to-end recognition of emotions in speech using CNNs followed by LSTM networks. The big difference in their method versus other deep learning algorithms is that they do not pre-select features before training the network. They introduce raw input to the system and let the black box choose the best representations for the features.

In their system, they create a segment of 6 s of raw audio first and preprocess it at a 16 kHz sample rate. They then pass the signal through the first convolution layer of 40 filters with a kernel of size 2 to bold the temporal features. The output of the first convolution layer is fed to pooling of size 2; the results are then fed to another convolution layer with a kernel of size 10 to smooth the temporal and extract the spectral features. Then it is fed to a pooling layer of size 20, reducing the dimensions of the data drastically. The reduced data for 6 s segments are then fed to a recurrent layer divided into 40 ms blocks; the LSTM layer is made of 128 cells [29]. Moreover, in 2019, Jianfeng Zhao et al. [30] proposed a framework based on two class of network blocks, a single layer convolutional block creating a local feature learning block (LFLB) and an LSTM block to learn global features. In their research, they have tried both 1-dimensional convolutions with the raw audio signal and 2-dimensional convolutions with Log Mel Spectrogram (LMS) features.

The LFLB block in their research is constructed by a convolution layer followed by a batch normalization (BN), an exponential linear unit (ELU), and a max-pooling at the end. Adding batch normalization by keeping mean and variance fixed helps every layer to be immune from large fluctuations from the previous layer, thus will improve the stability of the network; also by keeping input values in a limited range can help the model to converge faster and shorten training time [81].

The next block speeding up the learning in LFLB is ELU. D. Clevert et al. [82] introduced this block in 2016, ELU, similar to rectified linear units, tries to solve the vanishing gradient problem. Still, to improve the learning characteristics, ELU can have negative values that helps to push the mean activations closer to zero. Thus, increasing stability and speeding up the calculations by having smaller numbers in equations [82]. The last part in LFLB is the max pooling, which is widely used in almost every convolutional network. After extracting the low-level local features, the LSTM part has the job of extracting long-term contextual dependencies. LSTM has four components that can influence the state of the block: Input gate, Output gate, forget gate, and a cell with a recurrent connection to itself. The next stage in this proposed method is the fully connected layer for classification.

Their network in both cases of 1D and 2D is structured as follows, two layers of LFLB with 64 filters followed by two layers of LFLB with 128 filters followed by an LSTM layer with a kernel size of 256 and lastly, a fully connected layer. The convolution kernel in both implementations has a size of 3 and stride of one. To get the best results, they have run their experiments in both speaker-dependent and speaker-independent setting. In speaker-dependent settings, they have reached up to 92% accuracy for 1D and 95% accuracy for 2D networks. Additionally, in a speaker-independent setting, they have reached up to 62% accuracy for 1D and 82% accuracy for 2D networks.

In another research late in 2019, Xie et al. [83] introduced a system based on two layers of modified LSTMs with 512 and 256 hidden units, followed by a layer of attention weighting on both time dimension and feature dimension and two fully connected layers at the end. In their research, they have stated that humans’ attention on the whole stimuli is not balanced, and it has been shown incorporating this concept creates excellent results in image processing. Therefore, they have proposed a self-attention mechanism to the forgetting gate of an LSTM layer, which results in the same performance while reducing the computations.

Their change in coupled LSTM structure [84] reduces the forgetting gate required four training parameters to two parameters while experimentally does not affect the performance of the final LSTM mode. The input of the fully connected layer or any other block needs a predetermined length of features. In contrast, the LSTM output length varies based on the variable length of the input data. To solve this problem, they have proposed an attention weighting method for the output of all time steps and feature dimensions and then combining the results as a final output of the LSTM block.

They have experimented with five combinations of their proposed methods, LSTM with Time attention, LSTM with feature attention, LSTM with both time and feature attention, LSTM with modified forget gate, and LSTM with modified forget gate and time and feature attention. Additionally, as the results on their English speech dataset eNTERFACE, they have reached to 89.6% UAR accuracy in which they claim is the best result on that dataset.

LSTM networks have shown to be very effective in time series like data due to their pattern history memorizing capability. One of the default applications of such a system is speech emotion recognition. LSTM based systems are very well capable of learning the spectral features of the signal. When coupled with CNNs to learn the temporal characteristics of the signal, they can form a competent system to model and learn the samples.

All the mentioned exciting capabilities of LSTMs come with the cost of more process and exponential memory requirements. They also, similar to CNNs, need a vast number of training samples to tune their large number of variables.

### 5.3. Deep Learning Techniques for SER Enhancement

One major constraint in developing a powerful SER system capable of handling real-life scenarios is the mismatch of the train and test datasets and lack of generalization. To overcome these problems and improve the efficiency of the SER techniques, there are various methods incorporated as additions to the base blocks used in SER. In this section, we discuss recent achievements in deep learning that can be applied to overcome the limitations of SER.

#### 5.3.1. Autoencoders

Feature extraction is one of the essential tasks in classification, and one of its important objectives is to find a robust data representation in the presence of noise. Autoencoders are a group of unsupervised machine learning tools that can be utilized for this purpose. Generally, an autoencoder network has two components: encoder and decoder, which learns to construct a copy of the input to the output as closely as possible; therefore, the input dimensions and output dimensions are the same. The beauty of such a network is the hidden layer that describes the “code” to represent the data, i.e., code is a dense representation of the original data. In literature, several versions of autoencoders have been proposed. Among them, Variational Autoencoder (VAE), Denoising Autoencoder (DAE), Sparse Autoencoder (SAE), Adversarial Autoencoder (AAE) are very popular and useful in SER.

In 2018, Latif et al. [31] are the first researchers to propose VAEs to derive the latent representation of speech signals and use this representation to classify emotions in an intuitive way using deep learning. The dataset used in their experiment is IEMOCAP, the classifier is the LSTM network, and to condition the data representation on the emotion labels of the data, they experiment Conditional Variational Autoencoder (CVAE) where emotions are passed to the encoder as a condition variable.

In the same year, Eskimez et al. [32] utilized denoising autoencoder (DAE), variational autoencoder (VAE), adversarial autoencoder (AAE), and adversarial variational Bayes (AVB) as a tool of feature learning to improve the performance of speaker-independent CNN based SER systems. Since autoencoder is an unsupervised learning method, it does not suffer from a limited number of labeled data samples. In their work, they concluded autoencoder frameworks could be successful in increasing the F1 score and unweighted accuracy rating of automatic SER systems for SVM and CNN frameworks.

#### 5.3.2. Multitask Learning

In the development of a machine learning system, we optimize the model by improving a set of metrics on a single task. In this manner, we fine tune and tweak the model until its performance no longer increases. However, in this approach, we ignore information that comes from the training signals of related tasks, which might be useful in achieving the optimized model. The multitask learning (MTL) approach enables the model to generalize better on our primary task, through sharing representations between associated auxiliary tasks simultaneously.

In SER, speaker characteristics such as gender and age may affect how emotion can be expressed; therefore, they can be considered as Meta-information in MTL.

In 2017, Kim et al. [33], for the first time, use gender and naturalness as auxiliary tasks for deep neural networks in MTL manner. Their proposed method offers high-level feature representation where discriminative emotional clusters could be observed. They report the result of their practice using within-corpus and cross-corpus from six corpora. In their experiment, they used two architectures to investigate the effect of MTL on them, LSTM and DNN, along with learning on six corpora from simulated to natural datasets. Although in cross-corpora experiments, MTL outperformed STL and got a considerable gain in generalization, in within-corpora tests, their model improvement was not significant.

#### 5.3.3. Generative Adversarial Learning

Generative adversarial networks (GANs) have been considered as data augmentation, data representation, and denoising tools in deep learning since 2014 when Goodfellow et al. [85] proposed them for the first time to learn and mimic an input data distribution.

In 2018, Latif et al. [86] have used generative adversarial networks for the robustness of the SER system. They have shown how adversarial samples, generated by adding imperceptible noise to the legitimate samples, can attack SER systems and proposed the first adversarial attack on SER systems. Ultimately, their experiment results on IEMOCAP and FAU-AIBO have shown GAN-based defense against adversarial audio samples better confronts adversarial examples compared to adversarial samples and random noise training approaches.

General, also known as Vanilla, GAN [85], has two components, a generator neural network (*G*) and a discriminator neural network (*D*). The generator *G*(*z*) takes an input, *z*, a sample from probability distribution *P*(*z*), and generates synthetic data. On the other hand, the discriminator takes the information and determines whether the input data is real or generated. Finally, both networks reach an equilibrium in a way that they have a value function that one agent seeks to maximize, and the other tries to minimize as an objective function shown in the equation below:

(1) $$\min_{G}\max_{D}V\left(D,G\right)=\underset{x∼P(x)}{E}\left[\log D\left(x\right)\right]+\underset{z∼P(z)}{E}\left[\log\left(1-D\left(G\left(z\right)\right)\right)\right]$$

where D(x) and D(G(z)) are the probabilities that *x* and G(z) are inferred to be real samples by the discriminator.

In this framework, the GAN works unsupervised and independently from the class label of the real data. Succeeding, Conditional GAN [87] was proposed, which is based on the idea that GAN can be conditional by taking advantage of using Class labels and data from different modality or some part of the data. Unlike unconditional GAN, they can have more control over the generated data. In conditional GAN, the objective function of a two-player minimax game would be as follows:

(2) $$\min_{G}\max_{D}V\left(D,G\right)=\underset{x∼P(x)}{E}\left[\log D\left(x|y\right)\right]+\underset{z∼P(z)}{E}\left[\log\left(1-D\left(G\left(z\right)|y\right)\right)\right]$$

In this equation, “*y*” is the class label of the data.

In 2018, Sahu et al. [88] have investigated GAN’s usability in generating synthetic feature vectors for SER. In their experiment, they employed both Vanilla and Conditional GAN networks trained on the IEMOCAP dataset, and they have reported an improvement in SVM’s performance when real data is appended with synthetic data; however, the increase is not much. The result of the research confirms that to have a successful GAN framework, a large set of high-quality data is needed.

Later and in 2019, Chatziagapi et al. [89] utilized GAN as a conditioned data augmentation tool to overcome the SER systems’ data imbalance problem by generating synthetic spectrograms for the minority classes. During the experiment, GAN, fully convolutional architecture, approach beat Signal-based augmentation methods such as CP, CA, etc. and achieved relative performance improvement of by 5% to 10% on IEMOCAP and FEEL-25k datasets.

One of GAN approaches’ most important weaknesses is the convergence that highly depends on the data and initialization. However, to overcome such a limitation and for faster convergence, in their work, the GAN is initialized using a pretrained autoencoder. Moreover, to learn shared features between minority and majority classed, their proposed GAN is fine-tuned using both classes.

#### 5.3.4. Transfer Learning

Transfer learning can overcome the cross-domain’ challenge of SER, i.e., test corpora does not match train corpora. Song et al. [90] utilize transfer learning in cross-corpus speech emotion recognition task practicing dimension reduction and Maximum Mean discrepancy embedding optimization to get two adjacent latent feature spaces for the source and target corpora and SVM as classifier method. The experiment has been done utilizing EMO-DB with five emotion categories as source corpus and a Chinese emotion dataset with the same emotion categories as the test corpus. In two proposed models, principal component analysis (PCA) and local preserving projection have been used for dimension reduction. As a result, neutral has the best recognition rate, while happiness and fear have lower rates. However, compared to the automatic recognition approach, the proposed method achieved a better recognition rate.

In transfer learning, one significant restriction is the size of training data and test data, i.e., the number of training data for transfer learning should be so that you do not overfit the model. Moreover, since in reality, train and test datasets are not independent and identically distributed (i.i.d.); therefore, algorithms such as principal component analysis (PCA) and linear discriminant analysis (LDA) are performing poorly [91]. While the authors in [90] don’t discuss the aforementioned limitations, Song [91] studies the dilemma of “corpus bias” in speech emotion recognition offering transfer linear subspace learning (TLSL), generalized linear subspace learning, and transfer learning to consider the difference between train and test set, and application on several benchmark datasets. In the provided TLSL framework, for distance measurement, the nearest neighbor graph has been utilized as a regularization term to achieve shared feature representations and a feature grouping strategy to increase the transfer performance and bypass the negative transfer for cross-corpus speech emotion recognition. The framework has been applied to EMO-DB, eNTERFACE, and FAU Aibo databases in a cross-corpus manner in six possible ways, and the results show improvement in using TLSL compared to baseline methods since in the proposed approach. Unlike previous transfer learning approaches, the focus is not only on informative components of features, and less informative pieces are not neglected.

#### 5.3.5. Attention Mechanism

The attention mechanism for deep learning is another approach that recently achieved success within speech emotion recognition [83,92,93,94,95]. In typical deep learning methods for SER, all locations of a given utterance get equal attention; however, emotion is not uniformly distributed over the utterance for every sample. In the attention mechanism, the classifier regards the given samples’ specific locations based on the attention weights assigned to each part of the data, which contains an emotionally salient portion. Mirsamadi et al. [94], in an attempt to find more informative features about emotion instead of traditional low-level descriptors (LLD), and high-level statistical aggregation functions (HSF), utilized bidirectional LSTM with a weighted-polling method. This method was inspired by the attention mechanism, which allows the network to focus on emotionally salient parts of a sentence and ignore silent frames of utterance. The result of the study on the IEMOCAP dataset shows weighted pooling with local attention by balancing the short-term properties at the frame level with long-term aggregation at the utterance level, can improve SVM based SER’s performance compared to LSTM with mean-pooling. Later in 2019, Li et al. [95], utilizing attention mechanism and multitask learning, proposed a self-attentional CNN-BLSTM that improved the accuracy by an absolute of 7.7% compared to the multi-channel CNN [96] using the IEMOCAP dataset. The input of the framework is a speech spectrogram. To allow the CNN-BLSTM model to focus on the salient period of emotion, Self-attention has been applied, and ultimately, gender classification has been employed as an auxiliary task.

Late 2019, Xie et al. [83] proposed a system based on a modified LSTM. With their system, they had reduced the computational complexity by changing the forgetting gate of the LSTM in an attention gate. They also improved the efficiency of the system by applying the attention mechanism on both time and feature dimensions instead of just passing the output of the last iteration in LSTM.

In this research, the output of the LSTM, instead of being selected by the result of the last step, is being generated based on a number of the steps in time generated by an attention mechanism. Another similar approach is applied to the features, and lastly, the results are being fed to the fully connected layer for classification.

## 6. Discussion and Conclusions

In this section, we summarize the datasets, methods, and approaches in SER, followed by the identified challenges that led us to the future works. In the first part, we discuss the datasets and methods reviewed in the paper, and then we will talk about the challenges facing SER. After that, we will discuss some of the possible future works. Additionally, at last, we will conclude the paper. Table 3 shows a comparison between the techniques reviewed in this paper and the databases employed in those techniques. In this table, the black dots are the datasets used in each research.

As can be seen from Table 3, generally, EMO-DB was the most used dataset for older studies in the reviewed research. However, IEMOCAP has taken its place for more recently proposed methods as it has the larger sample pool more suited for training the deeper architectures. The third most used database in eNTERFACE as it was used in multiple SER challenges.

In Table 4, a concise comparison of all the methods reviewed in this paper has provided the research paper title, the year, the techniques used in the article, the feature employed in the proposed method, the databases used, and the highest accuracy obtained for each dataset.

To compare the proposed methods, we were interested in measures other than weighted or unweighted accuracy. However, only a few of the papers had provided other means, such as the F1 score. Some of the papers had multiple accuracies reported for different situations in which we took the best reported accuracy for each database used.

From Table 4, it can be seen that among the variety of features used for the SER task, MFCC was the most used feature representation. Additionally, openSmile features and feeding PCM or raw Audio data are used in the most recent research repeatedly.

In older studies, most of the methods were based on signal processing and traditional machine learning methods such as SVM. However, more recently, researchers are focusing on deep learning and neural networks improvement, which is directly related to the progress and advancement of hardware and software that allows researchers to employ and tune sophisticated networks such as LSTM, GAN, and VAE.

Additionally, reviewing the reported accuracies in Table 4, we can see some of the results are well above 90% accuracy. With more investigation, we can see that they all are using an older database such as EMO-DB and DES, which both have a minimal number of samples. Considering the size of the databases, we can suspect traces of overfitting being involved. In the case of Zhao et al. [30], the system has about 2,500,000 variables to tune based on 535 sentences in EMO-DB; the gap seems too big to be able to assume there was no overfitting.

**Figure 2:**
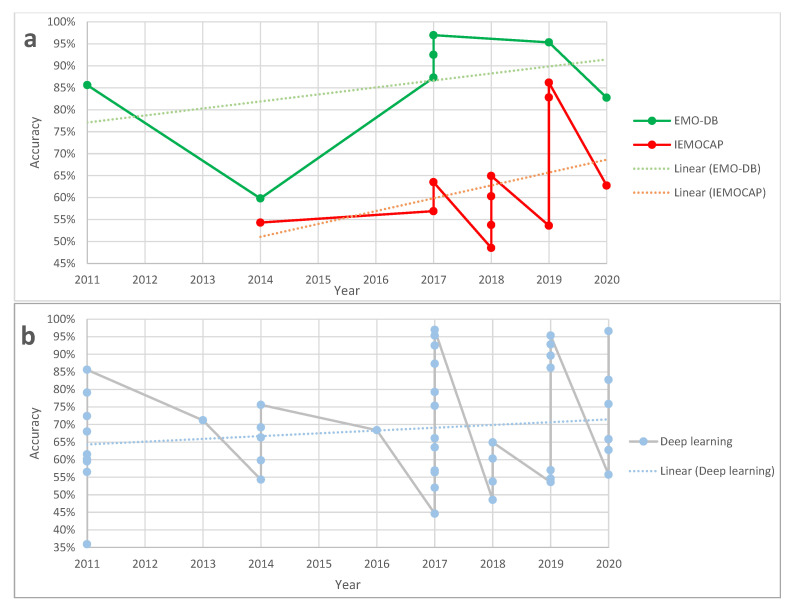
(**a**) Comparison of reported accuracies based on EMO-DB, IEMOCAP, and all databases used in deep learning methods and their trend lines. Dots in solid lines are showing all the accuracies reported and the dotted line shows the linear trendline of reported accuracies. (**b**) Aggregation of all the accuracies reported in deep learning methods.

Additionally, Figure 2a shows a comparison between accuracies reported in deep learning methods based on EMO-DB versus IEMOCAP, which we can see there is a clear separation between the accuracies published. Again, one reason could be the fact that EMO-DB has one degree of magnitude fewer number of samples than IEMOCAP, and using it with deep learning methods makes it more prone to overfitting.

Figure 2b shows an aggregation of all the accuracies reported in deep learning methods. As conveyed in this figure, relatively older deep learning methods, as a result of utilized feature extraction and classification methods that are using signal processing, except in few cases, have overall lower accuracies. However, recently the average accuracy has been increased. This also has been shown in both figures’ trend lines. The argument on overfitting and noise sensitivity of deep learning methods is still open, and in more recent years, studies have been done to address these issues too.

Another point reviewing accuracies and feature sets reported in Table 4 is that there is no apparent relationship between the complexity of the feature set and the accuracies reported, and the proposed methods have a significant role in the results. Incorporating similar databases Harar et al. [26], using EMO-DB, the feature set is just PCM samples of the wav file, and the accuracy is 96.97%. On the other hand, Song et al. [90], with a complex feature set, have reported an accuracy of 59.8%. Additionally, on the same databases, IEMOCAP, for two similarly, complex sets of features in Zhao et al. [30] have reported 86.16% accuracy while in Eskimez et al., 2018 [32] the accuracy is 71.2%.

### 6.1. Challenges

In SER, although there are progressions in methods and achieved accuracy; however, several restrictions still exist that necessary to be eliminated for a successful recognition system.

The main barrier is the availability of datasets well designed for deep learning tasks, meaning that they have a large enough pool of samples to be able to train deep architectures. In areas like image or speech recognition, there are databases with millions of samples such as ImageNet with 14 million and Google AudioSet with 2.1 Million samples. However, in SER, there are various databases, but with a limited number of samples.

Additionally, in most modern SER systems, semi-natural and simulated datasets are utilized that are acted in nature, not noisy, and far from reality. The systems trained on these datasets cannot be successful in real-world scenarios. Although real datasets are also available under license, however, they are from TV shows and call centers that parties are informed of the recording; therefore, they do not contain all emotion categories.

The other problem is the effect of culture and language on SER, where both factors affect the emotional feeling and receiving. A cross-language SER needs a set of features independent of these factors, and current feature extraction methods might not be successful.

In a similar context, another challenge with emotional speech databases is uncertainty in the annotation. As discussed before, in a task such as image recognition, a bicycle is always a bicycle; however, in an emotional speech, one may label an utterance as angry. In contrast, the other marks the same utterance as excited. This subjectivity in labeling both makes the task more complex and limits the possibility of mixing the databases and creating supersets of emotional data.

Furthermore, generally, datasets are made of discrete utterances of emotional speech while this is not guaranteed in real-life situations as usually, there are overlaps between the speech streams of speakers. Therefore, models designed based on discreet utterances would perform overwhelmingly poor in continuous speech situations.

In addition to continuity of the speech, also, changes in the flow of emotions are continuous and gradual, and we do not have sections of one emotion with a stopping point and changing over to other emotions. So the models designed for real-life situations should be able to handle in between and morphing emotions.

### 6.2. Future Directions

To solve the problem of SER, we need to address the challenges mentioned earlier. Additionally, one of the significant hurdles to SER is the limited size of the datasets. To solve this problem, one option is to create a deep learning friendly database, meaning a vast number of samples. This is a viable but costly method.

We also have the option of combining some of the datasets to create a superset. At the same time, this is possible; there could be problems because of different methods and techniques in creating different databases.

As a suitable solution, we suggest exploring the creation of an entirely synthetic dataset using generative techniques trained by available datasets. GANs structures would be an excellent candidate for such a system, as they have been used already and proven successful for other applications.

Another challenge that can be addressed in SER is the difference in emotion expressions in different languages. We believe using transformers, we can build a language-aware model that adapts to the language to classify emotions, and the same concept can be used for different accents in a language.

Furthermore, as we discussed laboratory-generated data and noise in real-life situations, we can use generator models as has been explored in the methods reviewed to create noisy samples and try to design a noise-robust model for SER.

Another point that we can improve the robustness of SER models is to create models that classify continuous speech emotions. For this reason, we can design architectures that are keeping a sliding window and measure the emotional content of the slide and decide based on that.

Additionally, to improve the SER model’s robustness, a similar concept can be employed to learn and classify not only fully emoted emotions but also the transition states of the feelings, and based on emotion transition models, we can gain more confidence in recognized emotions.

### 6.3. Conclusions

In this research, we have reviewed various emotional SER methodologies and the associated speech databases and compared them from different aspects. Among the databases we chose, there are two early and vastly used simulated databases, EMO-DB and DES. Additionally, we reviewed three newer, freely available simulated English databases. In addition to the simulated databases, we added IEMOCAP, a regularly cited semi-natural database, and VAM, one of the typically used natural databases.

Among all the databases compared, the newer ones tended to have a larger pool of data samples. The average duration of samples across the databases is 2.8 s, TESS being an outlier with 2.1 s average duration. Among the databases reviewed, TESS has very recently been cited to be used for the task of automatic speech emotion recognition yet. However, we examined this database, and we plan to use it in our future research, as it poses a more challenging task by having shorter utterances of emotional speech.

We have also reviewed several SER publications. In the selection of the papers, we tried to cover all the major deep learning techniques used for the task of SER, from DNNs to LSTMs to attention mechanisms. One widespread limitation in almost all the related works examined was the fact that they were only reporting the accuracy of the recognition as their performance measure, but statistically, accuracy by itself is not a comprehensive measure of the performance of a system.

The surge of new research on convolutional neural networks shows that they are capable of better solving the problem of emotion recognition by having higher low-level and short-term discriminative capabilities. The incorporation of LSTM networks and the introduction of deep convolutional LSTM structures has helped to take the solution to a new level and to give the network long-term memory to be able to identify long-term paralinguistic patterns. They have also shown higher capabilities of speaker-independent emotion recognition. Lastly, by the introduction of the attention methods, a new level of nonlinearity has been added to the classifiers that can, in turn, help in creating a more efficient system with fewer components. Future research could cover more robust and dataset-independent solutions to be able to move models closer to production in real situations.

## Figures and Tables

**Table 1:** Categories of emotional speech databases, their features, and some examples of each category. A black dot (•) means having a feature.

	Simulated	Semi-Natural	Natural
Description	created by trained speakers reading the same text with different emotions	made by asking people or actors to read a scenario containing various emotions	extracted from TV shows, YouTube videos, call centers, etc.
Natural emotions		•	•
Contains contextual information		•	•
Contains situational information		•	•
Discrete and separable emotions	•		
Single emotions at a time	•	•	
Widely used	•		
Standardized	•		
Easy to model	•		
Inter corpora results are comparable	•		
Large Number of emotions	•	•	
Used in real-world emotion systems modeling			•
Controlled privacy and copyright	•	•	
Examples	EMO-DB [55] DES [56] RAVDESS [57] TESS [58] CREMA-D [59]	IEMOCAP [60] Belfast [61] NIMITEK [62]	VAM [39] Call centers [42,56] AIBO [63]

**Table 2:** Statistics of the reviewed databases and their types of emotion. Availability of an emotion in a database is represented with a black dot (•) in the corresponding cell. The light gray section in the table shows the emotions shared between all the databases, and the darker gray section at the end of the table shows the 3 dimensions of continuous dimensional emotion representation.

	Berlin Database of Emotional Speech (EMO-DB)	Danish Emotional Speech Database (DES)	The Ryerson Audio-Visual Database of Emotional Speech and Song (RAVDESS)	Toronto Emotional Speech Set (TESS)	Crowd-sourced Emotional Multimodal Actors Dataset (CREMA-D)	Interactive Emotional Dyadic Motion Capture Database (IEMOCAP)	Vera am Mittag Database (VAM)
Number of emotions	7	5	8	7	6	9 emotions, 3 dimensions	3 dimensions
Number of samples	700	210	2496	2800	7442	1150	1018
Number of Speakers	10	4	24	2	91	10	47
Average Length	2.8 s	2.7 s	3.7 s	2.1 s	2.5 s	5 m	3.0 s
Anger	•	•	•	•	•	•	
Happiness	•	•	•	•	•	•	
Sadness	•	•	•	•	•	•	
Neutral	•	•	•	•	•	•	
Surprise		•	•	•		•	
Fear	•		•	•	•	•	
Disgust	•		•	•	•	•	
Boredom	•						
Calm			•				
Frustration						•	
Excited						•	
Valence						•	•
Activation						•	•
Dominance						•	•

**Table 3:** A concise comparison between all the reviewed deep learning methods sorted by year and the databases used. A black dot (•) in a cell means the corresponding database was used in the research mentioned at the bottom of the column.

Databases																									
EMO-DB	•	•				•			•	•	•		•					•	•		•			•	
DES	•		•																						
eNTERFACE			•								•		•								•		•		
ABC			•																						
SUSAS			•																						
SAL			•																						
SmartKom			•																						
AVEC			•		•																				
VAM			•		•																				
SEMAINE				•																					
SPD					•																				
IEMOCAP							•				•			•	•	•	•	•		•		•		•	
RECOLA								•																	
TEDLIUM2									•																
RML													•												
BAUM-1s												•													
AIBO											•				•						•				
LDC											•				•						•				
RAVDESS												•													•
Feel-25K																				•					
CASIA																							•		
GEMEP																							•		
CREMA-D																									•
SAVEE																									•
TESS																									•
Year	2005	2010	2011	2013	2014			2016	2017			2018						2019						2020	
Research	HMM, SVM [6]	SVM [17]	GerDA, RBM [22]	LSTM, BLSTM [28]	CRF, CRBM [24]	SVM, PCA, LPP, TSL [90]	DNN, ANN, ELM [23]	DCNN, LSTM [29]	CNN [21]	DCNN [26]	LSTM, MTL [33]	ANN, PSOF [19]	DCNN, DTPM, TSL [25]	LSTM, VAE [31]	GAN [86]	GAN, SVM [88]	LSTM, ATTN [94]	DCNN, LSTM [30]	CNN, VAE, DAE, AAE, AVB [32]	DCNN, GAN [89]	LDA, TSL, TLSL [91]	CNN, BLSTM, ATTN, MTL [95]	LSTM, ATTN [83]	DNN, Generative [76]	DCNN [79]

**Table 4:** A brief comparison of all the algorithms reviewed, containing the highest accuracy reported for each dataset, all the features used to train the system, methods used and if applicable, number of the layers in each method, and the title of the research.

Research Title	Methodology and Number of Layers	Features	Dataset and Accuracy
Speech Emotion Recognition based on HMM and SVM, Lin and Wei, 2005 [6]	HMM	1st and 2nd derivative of F0, 1st derivative of F1 2nd derivative of MBE4, 2nd derivative of MBE5 MFCC	DES: 99.5%
Speech Emotion Recognition Using Support Vector Machines, Chavhan et al., 2010 [17]	SVM	MFCC MEDC	EMO-DB: 93.75%
Emotion Recognition and Classification in Speech using Artificial Neural Networks, Shaw et al., 2016 [18]	ANN/1	Energy, Pitch, Formants 0 to 4 20 MFCCs	86.87%
Emotion recognition from Marathi speech database using adaptive artificial neural network, Darekar, and Dhande, 2018 [19]	ANN/1 PSO-FF	MFCC NMF Pitch	RAVDESS: 88.7%
Deep Neural Networks for Acoustic Emotion Recognition: Raising The Benchmarks, Stuhlsatz et al., 2011 [22]	GerDA RBM	Raw Signal ZCS, Signal energy Logarithmic F0 in Hz (Cepstrum and ACF) Exponentially smoothed F0 envelope Probability of voicing Spectral Energy various bands 25%, 50%, 75%, 90% roll-off point, centroid, flux, and rel. pos. max/min Mel-spectrum Band 1–26, 12 MFCCs	EMO-DB: 85.6% eNTERFACE: 72.4% ABC: 61.5% SUSAS: 56.5% AVIC: 79.1% DES: 60.1% SAL: 34.3% SmartKom: 59.5% VAM: 68.0%
Speech Emotion Recognition Using Deep Neural Network and Extreme Learning Machine, Han et al., 2014 [23]	DNN/3 ANN/1 ELM	MFCC Delta MFCC Pitch Period Harmonics-To-Noise Ratio (HNR)	IEMOCAP: 54.3%
Emotion Detection in Speech Using Deep Networks, Amer et al., 2014 [24]	CRF/3 CRBM	Spectrum	AVEC: 69.2% VAM: 66.3% SPD: 75.6%
Multi-Conditioning & Data Augmentation using Generative Noise Model for Speech Emotion Recognition in Noisy Conditions, Tiwari et al., 2020 [76]	DNN/3 Generative	HLDs (mean, standard deviation, skewness, kurtosis, extremes, linear regressions LLDs (zero-crossing rate (ZCR), RMS energy, F0, HNR, MFCCs)	EMO-DB: 82.73% IEMOCAP: 62.74%
A First Look Into A Convolutional Neural Network For Speech Emotion Detection, Bertero, and Fung, 2017 [21]	CNN/2	PCM	TEDLIUM2: 66.1%
Negative Emotion Recognition using Deep Learning for Thai Language, Mekruksavanich et al., 2020 [79]	DCNN/6	MFCC	SAVEE: 65.83% RAVDESS: 75.83% TESS: 55.71% CREMA-D: 65.77% THAI: 96.60%
Speech Emotion Recognition Using Deep Convolutional Neural Network and Discriminant Temporal Pyramid Matching, Zhang et al., 2017 [25]	DCNN (AlexNet)/8 DTPM/3 SVM	Log Mel-Spectrogram Delta Delta delta	EMO-DB: 87.31% RML: 75.34% eNTERFACE05: 79.25% BAUM-1s: 44.61%
Speech Emotion Recognition with Deep Learning, Harar et al., 2017 [26]	DCNN/10	PCM	EMO-DB: 96.97%
LSTM-Modeling of continuous emotions in an audiovisual affect recognition framework, Wöllmer et al., 2013 [28]	LSTM/1 BLSTM/1	Loudness, ZCR, Energy in 250–650 Hz, 1–4 kHz 25%, 50%, 75%, and 90% spectral roll-off points, flux, entropy, variance, skewness Psychoacoustic sharpness, harmonicity, 10 MFCCs F0 (SHS followed by Viterbi smoothing) Voicing, jitter, shimmer (local), delta jitter Logarithmic Harmonics-to-Noise Ratio (logHNR)	SEMAINE: 71.2%
Adieu Features? End-To-End Speech Emotion Recognition Using A Deep Convolutional Recurrent Network, Trigeorgis et al., 2016 [29]	DCNN, LSTM/4	PCM	RECOLA: 68.4%
Speech Emotion Recognition using deep 1D & 2D CNN LSTM networks, Zhao et al., 2019 [30]	DCNN, LSTM/5	PCM Log-Mel Spectrogram	EMO-DB: 95.33% IEMOCAP: 86.16%
Speech Emotion Classification Using Attention-Based LSTM, Xie et al., 2019 [83]	LSTM, DNN 5	F0, F0 envelope, ERMS noise and harmonics, and HNR Voicing probability, ZCS, Loudness and Delta Local jitter and shimmer, DDP jitter MFCC and Delta, Mel spectral and logMel bands LPC coefficients, Linear spectral pair frequency	eNTERFACE: 89.6% GEMEP: 57.0% CASIA: 92.8%
Variational Autoencoders for Learning Latent Representations of Speech Emotion: A Preliminary Study, Latif et al., 2018 [31]	VAE, LSTM 2, 4	Log-Mel Spectrogram	IEMOCAP: 64.93%
Unsupervised Learning Approach to Feature Analysis for Automatic Speech Emotion Recognition, Eskimez et al., 2018 [32]	CNN, VAE/5, 6, 4, 10, 5	Log-Mel Spectrogram	IEMOCAP: 48.54%
Towards Speech Emotion Recognition "in the wild" using Aggregated Corpora and Deep Multi-Task Learning, Kim et al., 2017 [33]	LSTM, MTL/3, 3, 2	F0, voice probability, zero-crossing-rate 12 MFCCs with energy and their first-time derivatives	EMO-DB: 92.5% eNTERFACE: 95.3% LDC: 56.4% Aibo: 52.0% IEMOCAP: 56.9%
Adversarial Machine Learning and Speech Emotion Recognition: Utilizing Generative Adversarial Networks for Robustness, Latif et al., 2018 [86]	LSTM, GAN/2	eGeMAPS features	Aibo: 64.86% IEMOCAP: 53.76%
On Enhancing Speech Emotion Recognition Using Generative Adversarial Networks, Sahu et al., 2018 [88]	GAN, SVM	1582-dimensional openSMILE feature space	IEMOCAP: 60.29%
Data Augmentation Using GANs for Speech Emotion Recognition, Chatziagapi et al., 2019 [89]	DCNN (VGG19), GAN/19	128 MFCCs	IEMOCAP: 53.6% Feel-25k: 54.6%
human–computer Using Transfer Learning, Song et al., 2014 [90]	PCA, LPP, TSL	12 MFCCs and Delta 8 LSF, Intense, Loudness, ZCR Voice probability, F0, F0 envelopes	EMO-DB: 59.8%
Transfer Linear Subspace Learning for Cross-Corpus Speech Emotion Recognition, Song 2019 [91]	LDA, TSL, TLSL	1582-dimensional openSMILE feature space	EMO-DB, eNTERFACE, Aibo: 54.61%
Automatic Speech Emotion Recognition using recurrent neural networks local attention, Mirsamadi et al., 2017 [94]	LSTM, ATTN/4, 3, 3, 4, 4, 4	257-dimensional magnitude FFT vectors F0, voice probability, frame energy, ZCR 12 MFCCs and Delta	IEMOCAP: 63.5%
Improved end-to-end Speech Emotion Recognition using self-attention mechanism and multitask learning, Li et al., 2019 [95]	CNN BLSTM, ATTN/2, 2	800 point STFT Mel scale spectrogram, Deltas, Delta deltas	IEMOCAP: 82.8%


## Data Availability

Not applicable.
